# Investigating Circular RNAs Using qRT-PCR; Roundup of Optimization and Processing Steps

**DOI:** 10.3390/ijms24065721

**Published:** 2023-03-16

**Authors:** Rares Drula, Cornelia Braicu, Sergiu Chira, Ioana Berindan-Neagoe

**Affiliations:** Research Center for Functional Genomics, Biomedicine and Translational Medicine, “Iuliu Haţieganu” University of Medicine and Pharmacy, 15 Victor Babeș Street, 400012 Cluj-Napoca, Romania

**Keywords:** circRNAs, RNAse R, miRNAs

## Abstract

Circular RNAs (circRNAs) have gained recent attraction due to their functional versatility and particular structure connected to human diseases. Current investigations are focused on the interplay between their ability to sponge smaller species of RNAs, such as microRNAs (miRNAs), thus influencing their regulatory activity on gene expression and protein templates. Therefore, their reported implication in various biological processes axis has resulted in an accumulating number of studies. While the testing and annotation methods of novel circular transcripts are still under development, there is still a plethora of transcript candidates suitable for investigation in human disease. The discordance in the literature regarding the approaches used in circRNAs quantification and validation methods, especially regarding qRT-PCR, the current golden standard procedure, leads to high result variability and undermines the replicability of the studies. Therefore, our study will offer several valuable insights into bioinformatic data for experimental design for circRNA investigation and in vitro aspects. Specifically, we will highlight key aspects such as circRNA database annotation divergent primer design and several processing steps, such as RNAse R treatment optimization and circRNA enrichment assessment. Additionally, we will provide insights into the exploration of circRNA-miRNA interactions, a prerequisite for further functional investigations. With this, we aim to contribute to the methodological consensus in a currently expanding field with possible implications for assessing therapeutic targets and biomarker discovery.

## 1. Introduction

Circular RNAs (circRNAs) are species of RNAs with limited coding potential [1,2] that are characterized by their specific enclosed loop architecture, particular biogenesis, and conservation [3]. Given their unique conformation, circRNAs were initially considered just results of aberrant splicing, with no endogenous function in eukaryotic cells, as the first circular RNA molecules were historically identified in plant viroids [4]. The advent of modern sequencing technologies allowed the annotation of these transcripts based on the parental transcript. It confirmed their abundance [5] and cell-specific expression pattern [6] in higher organisms. This accumulating evidence paved the way for the functional investigation of their physiological roles [7,8].

Regarding their biogenesis, circRNAs are derived from pre-mRNA transcripts and are generally composed of exonic sequences circularized in a process named “back-splicing” [9]. Specifically, back-splicing involves joining a 5′ splice donor with an upstream 3′ splice site [9].

In this model, internal exon circularization can be promoted by flanking long intronic sequences containing complementary sequences (such as *Alu* repeats) [10,11]. Yet, other combinations, such as exon-intron [12] or purely intronic circRNAs, do exist [13]. In addition to *Alu* repeats, which act as cis-promoting circularization elements [11], trans factors such as RNA-binding proteins (RBPs) have been described to promote RNA circularization, similar to the double-stranded RNA editing protein, ADAR1 [14]. It is crucial to mention that the competition between different RNA pairing elements from the intron sets of the pre-mRNA can dictate alternative circularization, resulting in multiple generated from the same original transcript, but with additional exon and/or intron composition [5,15]. While the consensus is that those circRNA transcript variants are less enriched than their linear counterparts, there is evidence that, in the case of several genes, the predominantly generated transcript is circular [5,7]. One of the main investigated functions of circRNAs is their competitive endogenous RNA (ceRNA) activity, acting as “sponges” for shorter single-stranded RNA molecules [7]. The primary examples, in this case, are microRNAs (miRNAs), short 19–22 nucleotide long non-coding RNAs, which are essential post-transcriptional regulators of gene expression [16,17]. The advent of modern sequencing and computational technologies allowed the discovery and annotation of thousands of novel circRNAs transcript variants [18,19,20]. This was based on multiple bioinformatic pipelines designed to facilitate their identification and annotation [19,21,22], and various databases which enabled their indexing [23,24]. On the other hand, more modern sequencing (such as Oxford Nanopore) techniques are addressing the limitations of current sequencing methods by extending read lengths to even full-length circRNA sequencing. Longer reads could improve the detection quality and address the variations imposed by circRNA isoform variations that could be overlooked by focusing solely on the BSJ region for the annotation [25].

**Figure 1:**
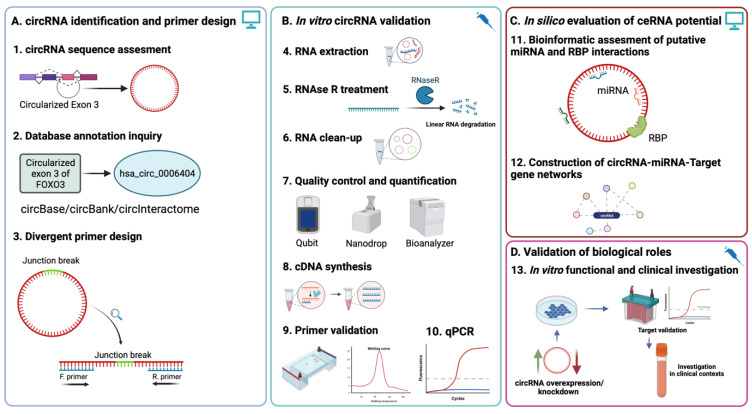
Experimental workflow of circRNA evaluation using qRT-PCR and downstream functional investigation. (**A**). In silico assessment of circRNA sequence and annotation based on parental mRNA region circularization is a prerequisite for convergent primer design based on junction sequence (Steps 1–3). (**B**). Appropriate RNA processing and the validation of previously designed primers ensure accurate and reproducible quantification of the circRNA of interest (Steps 4–10). (**C**). In silico investigation of ceRNA and RBP interactions can be indicative of potential regulatory roles of the circRNA via the construction of circRNA-miRNA-target gene networks. (**D**). In vitro functional validation of circRNA biological roles via knockdown/rescue experiments and validation of previously putative interactions can lead to the investigation of the circRNA in clinical contexts, either as a potential therapeutic target or biomarker.

The characterization of circRNA-miRNA-mRNA interaction networks became a popular model of study for the functional investigation of circRNAs, which have now been proven to be involved in multiple developmental processes [26], oncologic malignancies [27,28], and many others [16]. While there is still a high degree of variability and inconsistency in identification methods, validation using qRT-PCR has remained the gold standard in most functional studies of the circRNA [29]. Yet, many recent studies highlighted a need for more methodological consistency regarding the investigation methods. Here, we address these discrepancies by providing an in-depth description and step-by-step illustration of the stages required for the accurate characterization, validation, and data analysis of circRNAs using qRT-PCR to contribute to the methodological consensus. First, we will highlight the utility of database annotation and circRNA regarding sequence assessment based on parental transcripts and genomic coordinates, highlighting structural-specific aspects to be considered in the design and validation of convergent primers for qRT-PCR quantification. Then, we focus on several critical elements in optimizing processing steps, such as RNAse R treatment efficiency, analyzing circRNA enrichment following treatment, and possible correlations with the expression of their respective parental genes. Lastly, we will exemplify the utility of current online database resources for identifying potential miRNA targets, an important step for circRNA functional investigations. An overview of the workflow and experimental design is presented in Figure 1, highlighting both the bioinformatic and in vitro validation steps.

## 2. Results

### 2.1. Database Annotation, Composition, and Sequence Characterization of circRNAs

To exemplify the sequence and structural assessment required for the study of circRNAs, we chose to include four circular transcripts in our study, namely circular Forkhead box O3 RNA (circFOXO3), circular plasmacytoma variant translocation 1 (circPVT1), circular ArfGAP with Coiled-Coil, Ankyrin Repeat and PH Domains 2 RNA (circACAP2), and circular Anaphase-promoting complex subunit 2 RNA (circANAPC2). The first two transcripts are annotated in current databases. They have been investigated in several studies focusing on their functional interaction with miRNAs, yet they are not the main investigated variants [29,30,31,32]. We included them to offer an example for the investigation of validated circRNAs. For circFOXO3 and circPVT1, we utilized CircBase [33] and CircBank [34] to confirm the genomic location and annotated sequences for our circRNAs of interest based on the reposited sequencing data. On the other hand, while several versions of circANAPC2 [35] and circACAP2 [36,37,38,39] have been annotated, we employed a pseudo-reference-based approach for circRNA construction. We constructed the two pseudo-circRNAs from the exonic regions of their respective parental mRNAs gene using SnapGene. As such, for circANAPC2 and circACAP2, we included the back-spliced circularized exons 1 and 3 to 1 of their separate parental genes, resulting in two circular transcripts with lengths of 145 bp and 404 bp, respectively. Their detailed coordinates are presented in Table 1. For further reference, we included the complete characteristics of the utilized circRNA, such as their nucleotide and GC% composition (Supplementary Table S1). In addition, we calculated the probability for the formation of secondary structures based on the minimal free energy and internal complementary regions using mFold [40], which can be useful in assessing downstream interactions and applications (Supplementary Figure S2).

### 2.2. Junction Sequence Mapping and Primer Design

**Figure 2:**
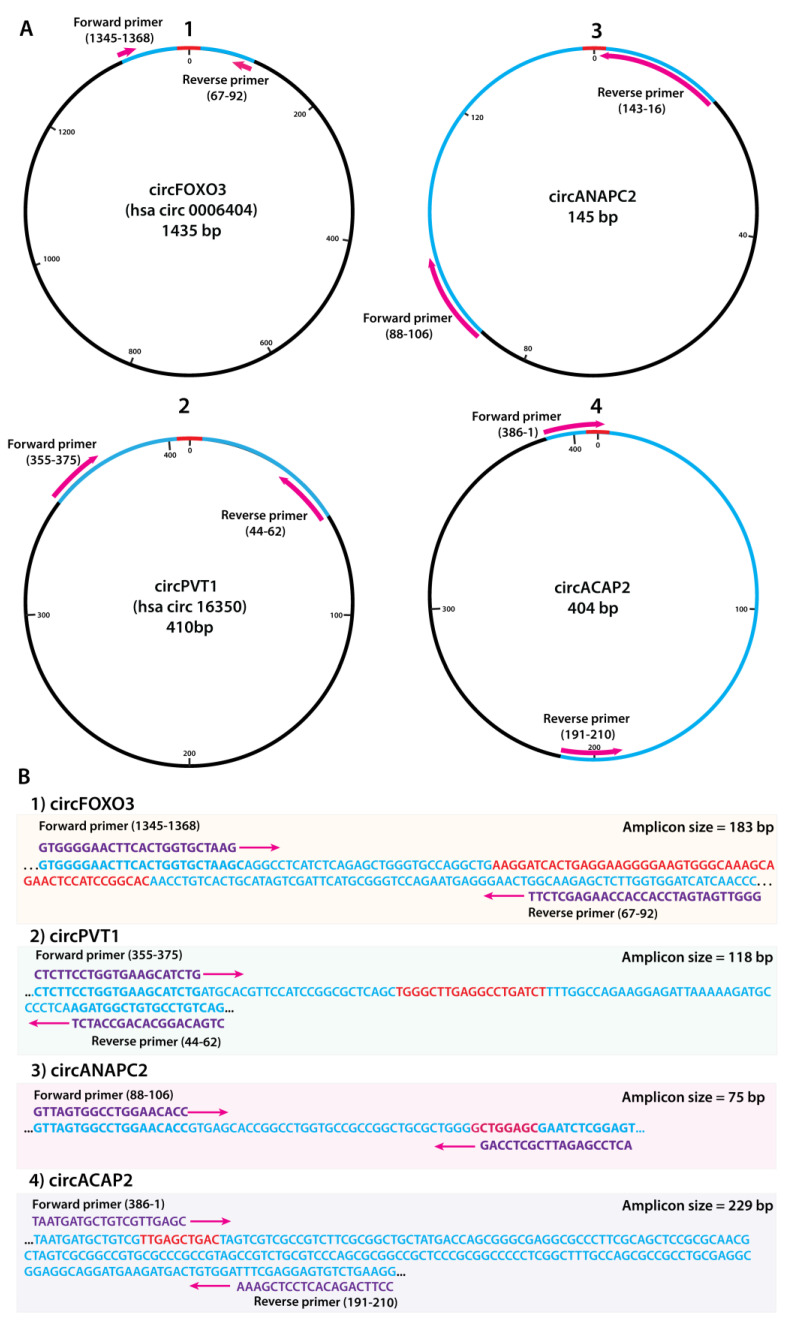
Map and sequence characteristics of the circular RNAs utilized in the study; (**A**) Circularized sequence maps of the circRNAs with highlighted primer binding positions and directions (in purple) and amplicons (in blue); (**B**) Amplicon sequence size and characteristics; primer binding positions and sequences (purple), complete sequence (blue) and highlighted junction sequence (in red) for all investigated circRNAs.

The key structural element to be considered when designing primers is the junction break region, which acts as the key distinguishing feature of circRNAs when compared to the linear cognate mRNAs. As such, the divergent primers should flank or overlap with the junction break in the circRNA sequence, as exemplified in Figure 2. As an estimated reference point for the primer design, we utilized the region bisected by the junction break, aiming for an amplicon size no larger than 250 bp. As an additional confirmation, amplification specificity has been assessed using the Primer design BLAST tool. All designed primers for the investigated circRNAs can be found in Supplementary Table S2.

### 2.3. Primer Validation

To assess the amplification specificity of the designed primers, we utilized agarose gel electrophoresis to validate the expected size of the amplicons. As circRNA expression can be cell-line specific, we generated a cDNA pool derived from equal amounts of RNA from multiple cell lines.

**Figure 3:**
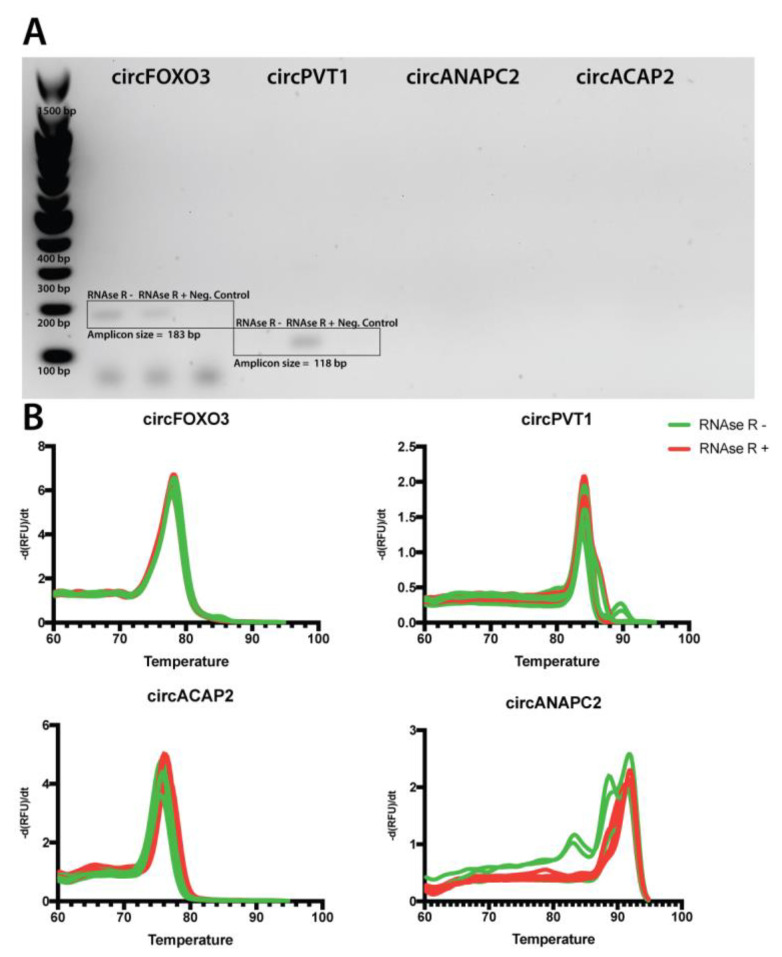
Validation of designed divergent primers for circRNA detection; (**A**). Agarose gel electrophoresis validation resulted in PCR circRNA amplified products of expected lengths for circFOXO3 (183 bp) and circPVT1 (118 bp), amplification products from circACAP2 and circANAPC2 could not be detected.; (**B**). Melting curve analysis indicates a single amplification product in all RNAse R treated samples in the case of all investigated circRNAs.

We first confirmed the specificity of the primers using touchdown PCR and demonstrated the size of the resulting amplicons using 2% EtBr-stained agarose gel electrophoresis. We confirmed that the resulting PCR products have the expected size in the case of circFOXO3 (183 bps) and circPVT1 (118 bps), with no other unspecific bands in either RNAse R− or R+ treated samples. Interestingly, the circPVT1 amplicon could not be detected in the RNAse R untreated sample, indicating a post-treatment enrichment of the transcript in the RNA pool. More so, for the pseudo-circRNAs, circANAPC2 and circACAP2, we could not observe any related products indicative of a superficial expression level, undetectable, using EtBr staining. As an additional confirmation, we evaluated the specificity of primers via the melting curve analysis resulting from the qRT-PCR using the cDNA from RNAse R treated and untreated RNA. This indicated a single melting peak in most reactions and replicated. At the same time, in the case of circPVT1 and circANAPC2, amplifications from the RNAse R+ cDNA (Figure 3B) produced more specific melting peaks when compared to the untreated samples. Overall, we confirm the amplification specificity of the primers and indicate that RNAse R treatment can influence circRNA amplification specificity.

### 2.4. RNAse R Treatment Optimization and RNA Quality Control

Linear RNA digestion using RNAse R is a commonly utilized step in processing the RNA samples for either sequencing or qRT-PCR investigations focused on circRNAs. In this case, we wanted to evaluate the linear transcript depletion efficiency and effects on circRNA detection. As such, we prepared individual reactions corresponding to four concentrations (0, 5, 10, and 20 units/reaction) of RNAse and three different time points (0, 60, and 120 min.) and proceeded to evaluate the effect of the various treatments on all investigated circRNAs and their parental linear transcripts.

**Figure 4:**
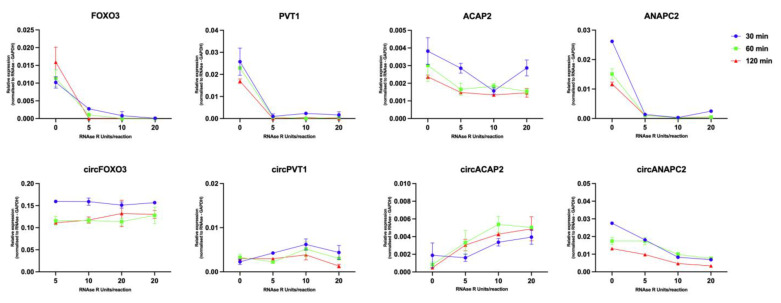
The effects of differential incubation time and enzyme concentration gradient RNAse R treatments on the average ΔΔCT (normalized using RNAse R mock treated GAPDH) levels of linear parental genes and the four investigated circRNAs. Each data point represents the replicate ΔΔCT value following the incubation at the specified time (legend) and enzyme concertation (on the *x*-axis).

As highlighted in Figure 4, we observed that the RNAse R significantly affects the stability of the linear transcripts compared to the circular RNAs. As expected, we observed a consistent degradation of all linear transcripts when compared to the mock-treated samples in the form of lower relative expression, correlated with the increased incubation time and amount of RNAse R. The parental linear transcripts displayed similar trends, with expression being dependent both on their enrichment levels in the RNA pool, but also their susceptibility to RNAse R degradation. The FOXO3, PVT1 and ANAPC2 mRNAs were efficiently degraded and displayed similar depletion trends. On the other hand, we noticed that ANAPC2 was more resistant to RNAse R when compared to the other linear transcripts, a factor often associated with either the presence of secondary structures or high G content in the RNA [41]. Interestingly, we observed that the maximum enzyme concentration acted as an inhibitory factor for the digestion efficiency, with most transcripts displaying higher depletion levels after adding 10 units of enzyme compared to 20 units. Referring to the effect of the RNAse R treatment on the detection of circRNAs, most circRNAs displayed minor modifications in the expression level. CircFOXO3 showed constant and relatively stable expression in all cases compared to the other circRNAs, regardless of treatment intensity, indicative of a high level of enrichment in the RNA pool. circPVT1 displayed a constant enrichment in the RNA pool gradually with the treatment intensity, with a slight decrease in the case of the 20 U reaction. Additionally, in the case of circACAP2, we noticed a constant increase in the expression trends following the RNAse R treatments. This effect was slightly mitigated again in the maximum enzyme input condition. On the other hand, the enrichment of circANAPC2 displayed a constant decrease following compared to the other circular transcripts, yet still not as significant as the degradation observed in its linear cognate. This indicates that the transcript is either susceptible to RNAse R activity or that the amplified product is not based on a circRNA.

Based on the indicated noticed expression trends, we considered an enzyme concentration of 10 units/reaction and an incubation time of 1h as the optimal treatment conditions for linear RNA depletion. In this way, we were also considering the background RNA degradation associated with longer incubation time noticed in the case of most transcripts, based on the lower detection levels in the samples incubated for 60 and 120 min when compared to the ones which were incubated for 30 min. We also observed a dose-dependent aspect in which the highest amount of RNAse R, namely 20 U/reaction, can have inhibitory effects on the reaction. Referring to the quality of the post-processing RNA, the RNAse R treatment utilizing this time/dose combination (10 units/reaction for 1 h) did not have any impact on the quality of the total RNA after appropriate cleanup, as indicated by the RNA integrity number (RIN) from the Bioanalyzer analysis. Yet, as expected, there was a significant decrease in RNA concertation in the treated samples, related to the degradation of the linear transcripts (Supplemental Figure S3, Supplemental Table S3).

### 2.5. Assessing Enrichment of circRNA following RNAse R Treatment

For a more accurate assessment of the enrichment of the investigated transcripts following RNAse R treatment, we calculated the circRNA expression data using two different methods. First, we utilized a circRNA-specific method based on the ratios (in percentages) between the averaged CTs in the untreated (representing 100%) and treated samples, as indicated by Panda and Gorospe [42]. Then, we adapted to the more rigorous ΔΔCT calculation method showcased by Taylor et al. [43] by utilizing the RNAse R mock-treated samples for calculating the normalization factor to highlight the amplitude of the post-treatment enrichment.

**Figure 5:**
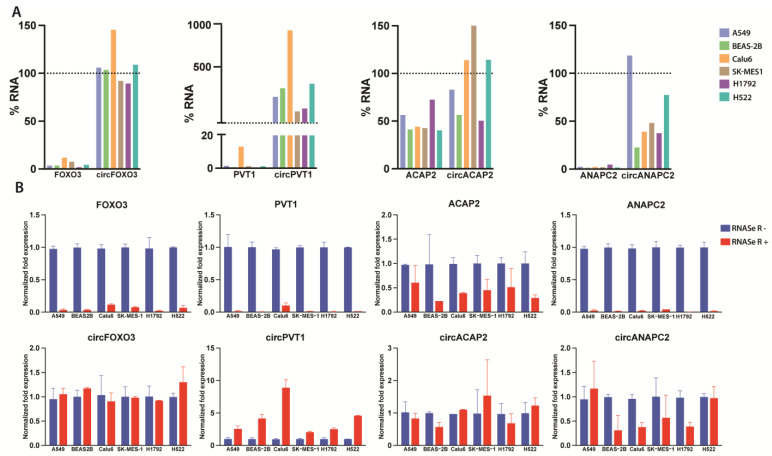
Overview of post-RNAse R digestion enrichment levels; (**A**) Percentages based on paired treated vs. untreated sample of the RNAse R treated samples; (**B**) Normalized mRNAs and circRNA DDCTs fold expression (normalized using the relative quantity (ΔCT) factor of the mock RNAse R treated GAPDH), based on the Taylor et al. [43] ΔΔCT method.

First, we wanted to validate whether the expression levels of the circRNAs are reflected by their parental gene’s expression level, an idea consistent with our reported comparative expression level analysis. As such, we proceeded to investigate the enrichment percentages as a form of direct quantification of the effect of the RNAse R treatment (10 units/reaction) level in multiple lung cancer cell lines (Figure 5, Supplemental Table S4). Overall, we observed consistently elevated expression of both mRNA and derived circRNA in the case of only several samples, such as in H522 cells for circFOXO3 and circACAP2 and SK-MES1 cell line for circPVT1, indicating that, besides expression patterns, the ratio between circRNA and parental transcript is primarily cell-type specific (Supplemental Figure S4).

Following the enrichment assessment, we report a significant and consistent transcript depletion in the case of all linear genes. While varied ratios can be observed in many instances, we assume they can be based on the cell-line-specific expression patterns reflected in their basal expression levels (Supplemental Figure S3B). Additionally, we confirmed the previously observed resistance of the ACAP2 transcript to the RNAse R treatment, as it maintained a higher percentage of transcript integrity than the other investigated genes. Out of all the circRNAs, circFOXO3 and circPVT1 displayed the highest levels of enrichment, in line with the previously shown trends from the gradient treatments’ samples. More so, circPVT1 indicates a significant enrichment following RNAse R treatment in all samples. In the case of the other two investigated circRNAs, circANAPC2 and circACAP2, enrichment higher than 100% of the untreated sample was observable in several samples derived from several cell lines. Yet, both transcripts display much higher resistance to RNAse R when compared to their linear parental cognate.

As raw C_T_ values taken out of context cannot be used as reliable data, only as a first indication, we utilized additional calculation methods [43]. Factoring in aspects such as the dynamic nature of circRNAs expression and discordance regarding normalization methods, we adapted an extensive ΔΔC_T_ calculation method, which included multiple normalization steps, factoring RNA input based on reference gene, per sample, and biological group normalizations. Given the susceptibility of the GAPDH mRNA and other normalizing transcripts to RNAse R, we utilized the reference gene values for the mock-treated RNAse R sample instead of its corresponding values. While we noticed similar trends (Figure 5B) to the first CT average-based enrichment calculation, normalization steps are crucial for contextualizing the illustrated data and strengthening its replicability.

### 2.6. Database Prediction of Interacting miRNAs and Construction of circRNA-miRNA-mRNA Networks

**Figure 6:**
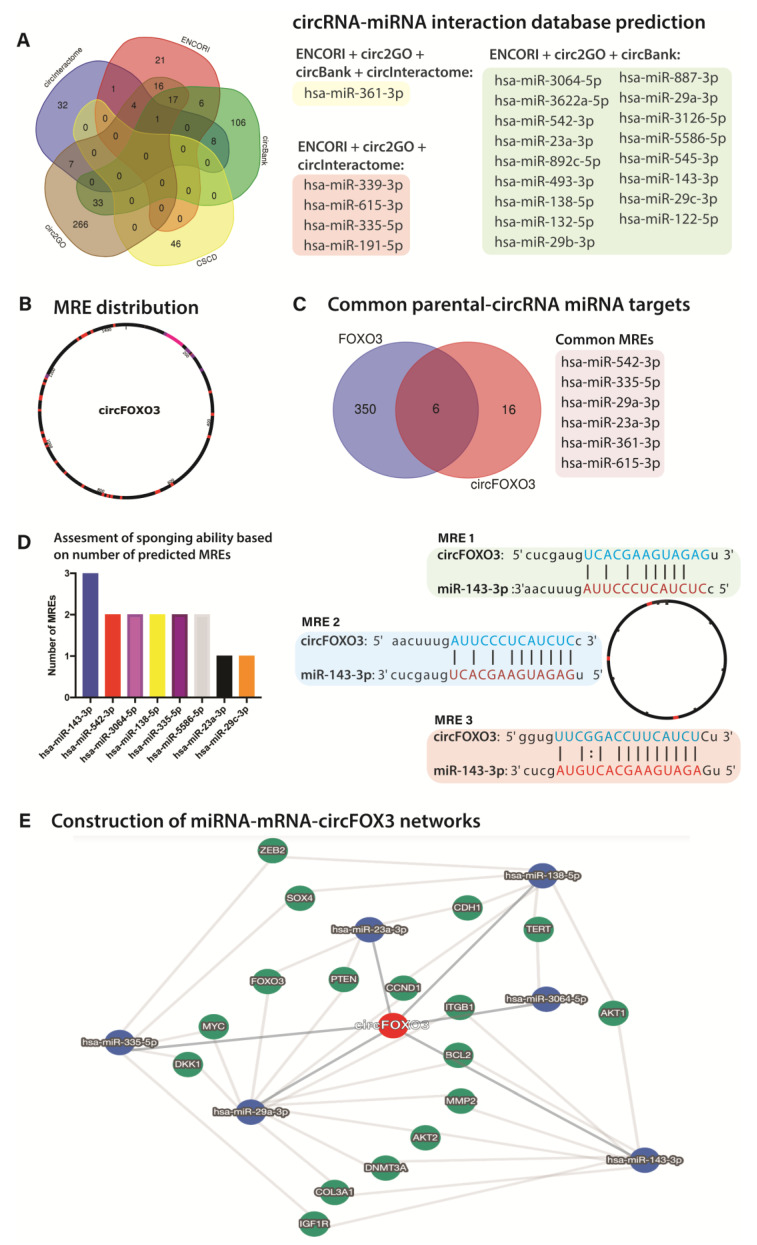
miRNA interaction-based database construction of circFOXO3-miRNA-target gene networks (**A**) Venn diagram depicting the overlapped putative miRNA candidate list obtained from circRNA databases; (**B**) Example of distribution of MREs in the sequence of circFOXO3; (**C**) Venn diagram depicting the overlapping miRNAs potentially competitively interacting with both the linear FOXO3 mRNA and circFOXO3; (**D**) Number of MREs for each miRNA in the sequence of circFOXO3 (left) and exemplified distribution of MREs for miR-143-3p in the sequence of circFOXO3 (**E**) Exemplified constructed circRNA-miRNA-target gene focusing on the primary miRNAs interacting with circFOXO3 and their putative target genes.

As the current focus on circRNAs is based on their miRNA sponging ability, we chose circFOXO3 as a relevant example to highlight all the steps of assessing miRNA interactions using the available circRNA databases. Given its sequence length, database annotations, and overall prevalence in circRNA studies, multiple putative circFOXO3 interacting miRNA datasets are available. We selected the most representative circRNA database with a comprehensive list of human circRNAs. We predicted binding miRNA tools, namely ENCORI (Starbase) [44], circBank [34], CSCD [45], circ2GO [46], and CircInteractome [47]. The characteristics regarding utilized annotation and tools in these databases are presented in Supplemental Table S5. We generated an interaction list of 22 miRNAs occurring in at least three overlapping datasets out of the five inquired (Figure 6A), with only has-miR-361-3p being common in 4 and 15 additional results from the overlapping of at least three databases. The MRE (miRNA response elements) distribution in the circRNA transcript can indicate the presence of binding hotspots in the circRNA sequence consisting of either multiple overlapping MREs or conserved binding situses for miRNA families. We investigated the MRE distribution for the selected relevant miRNAs in the circFOXO3 sequence (Figure 6B). In the case of circFOXO2, the MREs display an overall uniform distribution pattern, with a small agglomeration of 100 bp upstream and downstream of the 200 bp mark.

Determining the overlapping circRNA-parental mRNA MREs can highlight a competitive binding potential for the specific miRNAs. Yet, the shared sequence elements (and lack thereof) between the circRNA and the parental gene must be considered. Canonically, miRNAs bind with the 3′ UTR of their targeting mRNA to determine any regulatory effect. In our case, given the exonic sequence composition of circFOXO3, the vast majority of MREs present in the FOXO3 mRNA would not be present (i.e., MREs from the 3′UTR) in circFOXO3, and the number would be restricted by the number of MREs present exclusively in the second exons of the circRNA mRNA. After comparing the circFOXO3-miRNA putative interaction list with the linear cognate FOXO3 targeting miRNAs, out of the total 22 circFOXO3 putative miRNAs, 6 were indicated to have a competitive binding potential for both circFOXO3 and the FOXO3 mRNA (Figure 6C). Assessing the presence of multiple MREs for the same miRNA can indicate the susceptibility to the sponging abilities of the circRNA. For example, out of the selected miRNAs, several are shown to have multiple MRE harbored in the circFOXO3 transcript, implying a possible stronger sponging ability, such as miR-143-3p. As highlighted in Figure 6D, the-miR-143-3p presents three putative binding situses distributed in the circFOXO3 sequence, while miR-29c-3p- has only one binding site. To understand the biological roles and potential functions of the circRNA-miRNA interactions, a restrictive circRNA-miRNA-target gene interaction network was generated based on the relevant identified miRNAs (Figure 6D).

## 3. Discussion

Circular RNAs are currently being intensely investigated, given their functional versatility and regulatory potential of miRNA-target gene interactions, gaining significance as potential targets in pathological processes [48]. Therefore, the consistency and replicability of circRNA investigation methods are crucial for reaching a consensus regarding their potential clinical or fundamental research applications [29]. The constant development of bioinformatic pipelines seeking to assemble and annotate circRNAs from RNAseq datasets accurately generates extensive lists of likely circular transcripts, which require experimental validation. While novel full-length sequencing methods could drastically facilitate detection and address annotation inconsistencies, the proposed transcripts would still require additional qRT-PCR for the downstream functional validation [25]. Here, we aimed to contribute to this consensus by highlighting a standardized workflow to validate and investigate potential circRNAs (Illustrated in Supplemental Figure S1). Firstly, we adopted an exploratory approach by selecting validated and pseudo-referenced circRNAs to highlight the validation steps. Although circFOXO3 Field [30,31,32] and circPVT1 Field [49,50,51] have been previously investigated in malignant contexts, we utilized them as appropriate candidates for method optimization, given their expression levels for detection and sequence length.

We focused on the importance of the junction sequence as the crucial circRNA distinctive factor in the primer design rationale. Following the bioinformatic characterization and primer validation for the selected circRNAs, we experimentally optimized the main RNA processing steps focusing on starting concentration, RNAse treatment intensity, and RNA quality control. Based on our experience, we do not recommend starting with less than 1 ug of total RNA to account for the losses associated with the RNAse R treatment and further purification step. Through the RNAse R optimization step, we highlighted the inhibitory effect of increased RNAse R concentrations upon the degradation of linear transcripts. We indicate that an optimal incubation time and concentration are reflected in the efficient degradation of linear RNAs and the enrichment of stability of circRNA in the investigated sample. This can be seen in the comparative enrichment analysis, which highlights the importance of including the untreated control samples for assessing the RNAse R treatment’s efficiency in enriching the circRNA fraction ratio out of the total RNA pool. Regardless, the comparative enrichment analysis’s full extent can only be based on contextualized normalized data, which can consider the dynamicity of circRNA expression in the total RNA pool. In our case, we noticed sample and circRNA-specific variations in enrichment levels. High levels of post-treatment enrichment, such as circPVT1, can be attributed to its increased dilution in the total untreated RNA pool, resulting in a lower initial detection level.

The two unannotated pseudo-circRNAs (circANAPC2 and circACAP2) displayed lower enrichment levels in several samples, which we assume to be associated with (a) their deficient expression levels and (b) the possibility that they are not bonafide circular transcripts. Higher RNAse R concentrations have been reported to affect circRNA stability, confirming the requirement for transcript-specific optimization of the RNAse R input [52]. Yet, both displayed significant resistance to RNAse R digestion compared to their linear cognates. As a particularity, the ACAP2 mRNA showed considerable resistance to RNAse R in multiple cases. Resistance of linear transcripts to RNAse R treatments has been previously reported, especially in the case transcripts characterized by a high number of G-quadruplex (G4) [41].

Additionally, since RNAse R is a 3′->5′ exonuclease, its efficiency can be limited by multiple transcript-specific factors. Highly structured 3′ ends with overhangs shorter than 5 nucleotides, short polyA tails, and an overall prevalence of secondary structures can hinder its efficiency. Therefore, optimization for the specific removal of these resistant transcripts is required for an efficient depletion. To address this limitation, additional steps to the RNAse R digestion methods were developed, such as polyadenylation of resistant transcripts (post-digestion) and their subsequent depletion using polyA removal kits can efficiently deplete resistant transcripts [53]. Overall, we indicate that the RNAse R digestion step is necessary and requires optimization preceding the downstream investigation of a specific circRNA.

The principal investigated the function of circRNAs as the biological effect of their sponging interaction with specific miRNA, which in turn ablates their regulatory activity upon other RNAs. Focusing on circFOXO3, we exemplified a thorough approach using the online databases tools to select the relevant putative miRNA candidates for further functional validation. The databases rely on mostly different alignment algorithms, so we recommend choosing the miRNA target candidates highlighted in multiple tools provided lists. We emphasized the differential binding ability of specific miRNAs that display numerous binding sites in the circRNA sequence, presenting a higher critical potential, such as miR-143-3p. Even if the literature on circFOXO3 is still restrictive, the interaction between miR-143-3p and circFOXO3 has been previously investigated [31]. The two transcripts are involved in a tumor-promoting regulatory axis in the case of gastric cancer involving USP44, confirming the solid binding interaction. Exploring and validating the biological roles of circRNA-miRNA-Target networks is a crucial aspect of investigating the functionality of circRNA in different pathological contexts. Also, miRNA interactions can only be valid if accompanied by appropriate methods, such as Ago-RNA Immunoprecipitation (AGO-RIP) [54]. Although qRT-PCR provides strong sustaining evidence [52,55], it is crucial to mention that confirming the existence of a circRNA is conditioned by additional validation methods. Most commonly, the Northern Blot [56], Sanger sequencing of amplification products, or new generation sequencing [22] focused on the sequence validation of junction break regions are recommended.

As such, selecting these methods is case-specific and based on experimental design and available resources. In our case, we focused on the RNAse R and qRT-PCR optimization of the validated transcripts while also including two not yet validated circRNAs, as an initial screening of potential transcripts based on their sensitivity to RNAse R treatments.

## 4. Materials and Methods

### 4.1. Cell Lines

Immortalized human bronchial epithelial cells (BEAS-2B) and human lung cancer cell lines A549, Calu6, H522, H1792, SKMES-1 were purchased from the American Type Culture Collection (ATCC, Manassas, VA, USA); BEAS-2B cells were cultured in BEBM serum-free medium (CC-3170, Lonza, Verviers, Belgium), A549 cells were cultured in F12-K media supplemented with 10% fetal bovine serum and Calu6, H522, H1792, and SKMES-1 cells were cultured in RPMI-1640 medium (Hyclone, Logan, Australia) containing 10% fetal bovine serum at 37 °C and 5% CO_2_ under saturated humidity.

### 4.2. RNA Extraction

Cell lines were cultured until 70–80% confluency, washed, and dissolved in TripleXtractor (Grisp, Porto, Portugal). Total RNA was extracted using the phenol-chloroform method, and the resulting pellet was resuspended in RNAse/DNAse-free water. DNAse treatment was carried out utilizing TURBO DNA-free™ Kit (Life Technologies, Carlsbad, CA, USA) following the manufacturer’s instructions. The resulting RNA was quantified using NanoDrop 3000 and Qubit™ RNA High Sensitivity Assay Kits (Thermo Fisher Scietific, Waltham, MA, USA) to assess the amount and quality.

### 4.3. RNAse R Treatment

Dilutions of 1 ug of total RNA were prepared and subjected to RNAse R treatment to deplete linear RNAs. The selected concentration of RNAse R (10 U/µL) (Applied Biological Materials, Vancouver, BC, Canada) was added to the RNA sample with 2 µL of 10× RNAse R Buffer (0.2 M Tris–HCl (pH 8.0), 1 mM MgCl_2_, and 1 M KCl, NaCl or LiCl) in a total reaction volume of 20 µL and incubated at 37 °C for the mentioned time. For each RNA sample, a corresponding control sample was prepared in which water was added instead of RNAse R.

### 4.4. RNA Clean-Up and Quantification

The resulting RNA from the RNAse R and untreated samples were purified using RNeasy Kit (Qiagen, Germantown, MD, USA), following the manufacturer’s instructions. Total RNA was eluted in 20 µL of nuclease-free water. The resulting RNA was quantified using Nanodrop 3000 and Qubit fluorimeter (both produced by Thermo Fisher Scietific, Waltham, MA, USA). Qubit measurement was executed using Qubit™ RNA High Sensitivity (HS) assay kit using 1 µL of the RNA sample, 198 µL of kit Qubit working solution, and 1 µL of the provided fluorophore. RNA amount was normalized based on the standard curve and input. The quality of the RNA was evaluated using the Bioanalyzer 2100 (Agilent Technologies, Santa Clara, CA, USA) RNA 6000 Nano kit using the manufacturer’s instructions. Samples with a RIN > 8 were considered adequate for further analysis.

### 4.5. cDNA Synthesis

A total of 100 ng (based on amounts indicated from Qubit quantification) was reverse transcribed into cDNA using AzuraFlex^TM^ cDNA Synthesis Kit (Azura Genomics, Raynham, MA, USA) in a 20 µL reaction volume in the Veriti™ 96-Well Fast Thermal Cycler (Applied Biosystems, Waltham, MA, USA). Kit-provided random hexamers instead of oligo dT primers were utilised to amplify both circular and polyadenylated RNAs non-discriminately.

### 4.6. Primer Validation PCR and Agarose Gel Electrophoresis

Corresponding PCR amplification reactions using cDNA from RNAse R treated and untreated were carried out using Phusion™ High-Fidelity DNA Polymerase following the manufacturer’s instructions. circRNA primers were synthesized by RealTimePrimers (https://www.realtimeprimers.com, accessed on 1 April 2022). The primer annealing temperature was assessed using a temperature gradient approach based on the calculated primer TM. Primer sequences can be found in Supplemental Table S1. Tubes were placed in the Veriti™ 96-Well Fast Thermal Cycler. Samples and appropriate 100 bp DNA size ladder (New England Biolabs, Ipswich, MA, USA) were mixed with loading dye and loaded on a 2% Agarose EtBr containing gel. The resulting bands were compared based on the expected amplicon size.

### 4.7. qRT-PCR

circRNA and linear gene expression were evaluated using AzuraView™ GreenFast qPCR Blue Mix LR (Azura Genomics, Raynham, MA, USA) using 1 µL of 1:5 diluted cDNA from both RNAse R treated and untreated samples (with either 30 or 100 ng of RNA input). qRT-PCR assays were performed on ViiA 7 Real-Time PCR System using the recommended amplification protocol (45 cycles of 30 s at 95 °C, 30 s at 55 °C, and 30 s at 72 °C). The total reaction volume was 10 µL, and GAPDH was used to normalize and evaluate RNAse R treatment efficiency. Technical duplicates were utilized for all reactions. Utilized primer sequences are presented in Supplemental Table S1. The melting curve step was included in all amplification reactions based on the instrument’s temperature gradient settings.

### 4.8. Data Analysis

Reference circRNA sequences and IDs were downloaded from circInteractome (https://circinteractome.nia.nih.gov, accessed on 1 April 2022) and circBank (www.circbank.cn accessed on 1 April 2022). Linear transcript sequences were downloaded from Ensembl (www.ensembl.org accessed on 1 April 2022), and the exonic circRNAs were constructed using SnapGene Software (www.snapgene.com accessed on 10 April 2022). The divergent primer design tool in the circInteractome tool was utilized for the primer design. Putative binding miRNAs were assessed using the online tools provided by the circInteractome (https://circinteractome.nia.nih.gov accessed on 15 May 2022), ENCORI-Starbase (https://starbase.sysu.edu.cn accessed on 15 May 2022), Cancer-Specific circRNA Database (http://gb.whu.edu.cn/CSCD accessed on 15 May 2022), and Circ2GO (https://circ2go.dkfz.de accessed on 15 May 2022) online databases. Venn diagrams were generated using the following online tool (https://bioinformatics.psb.ugent.be/webtools/Venn/ accessed on 15 May 2022). CircRNA-miRNA-Target gene networks were constructed using miRTargetLink 2.0 (https://ccb-compute.cs.uni-saarland.de/mirtargetlink2 accessed on 20 May 2022). Data representation was carried out using GraphPad prism 9. All data are reported as the mean ± standard deviation (SD). Statistical comparisons between two groups are made using Student’s two-tailed unpaired *t*-test, and *p* < 0.05 is considered statistically significant.

## 5. Conclusions

CircRNAs have recently emerged as important players, along with miRNAs, in the dogmatic shift of post-transcriptional regulation of gene expression. While the recent influx of emerging studies has established their relevance in a plethora of mechanisms, it has also outlined the methodological shortcomings and complexity of circRNA investigations. This study provides a step-by-step overview of the critical design and optimizing steps in circRNA investigations. Our results emphasize that optimizing the prerequisite RNAse R treatment step and considering RNA quality is crucial for adequate detection of circRNAs by RT-qPCR. Such methodological developments, along with correct bioinformatic sequence assessment and consensus regarding circRNA nomenclature, will facilitate and accelerate circRNA investigations, thus offering a clear understanding of their regulatory roles and therapeutic utility.

## Figures and Tables

**Table 1:** CircRNA annotation, genomic location, and characteristics.

CircRNA	Parental Gene	CircBase ID	Structure	Genomic Location	Length
circFOXO3	FOXO3	hsa_circ_0006404	Exon 2	chr6:108984657-108986092	1435 bp
circPVT1	PVT1 lncRNA	hsa_circ_0009143	Exon 2	chr8:128867400-128903244	410 bp
circANAPC2	ANAPC2	Unannotated variant	Exon 1	chr9:137188416-137188560	145 bp
circACAP2	ACAP2	Unannotated variant	Exon 3-1	Chr3:195442795-195381903	404 bp

## Data Availability

All data can be available upon reasonable request.

## Supplementary Materials

The following supporting information can be downloaded at: https://www.mdpi.com/article/10.3390/ijms24065721/s1.
