# Supplementary material for: Investigating Circular RNAs Using qRT-PCR; Roundup of Optimization and Processing Steps
Source: Int J Mol Sci. 2023 Mar 16;24(6):5721. doi: 10.3390/ijms24065721 (PMC10056686; doi:10.3390/ijms24065721)
Supplement: Supplementary file 1 [file ijms-24-05721-s001.zip › ijms-2208076-supplementary.pdf]

Supplementary Figures and Tables

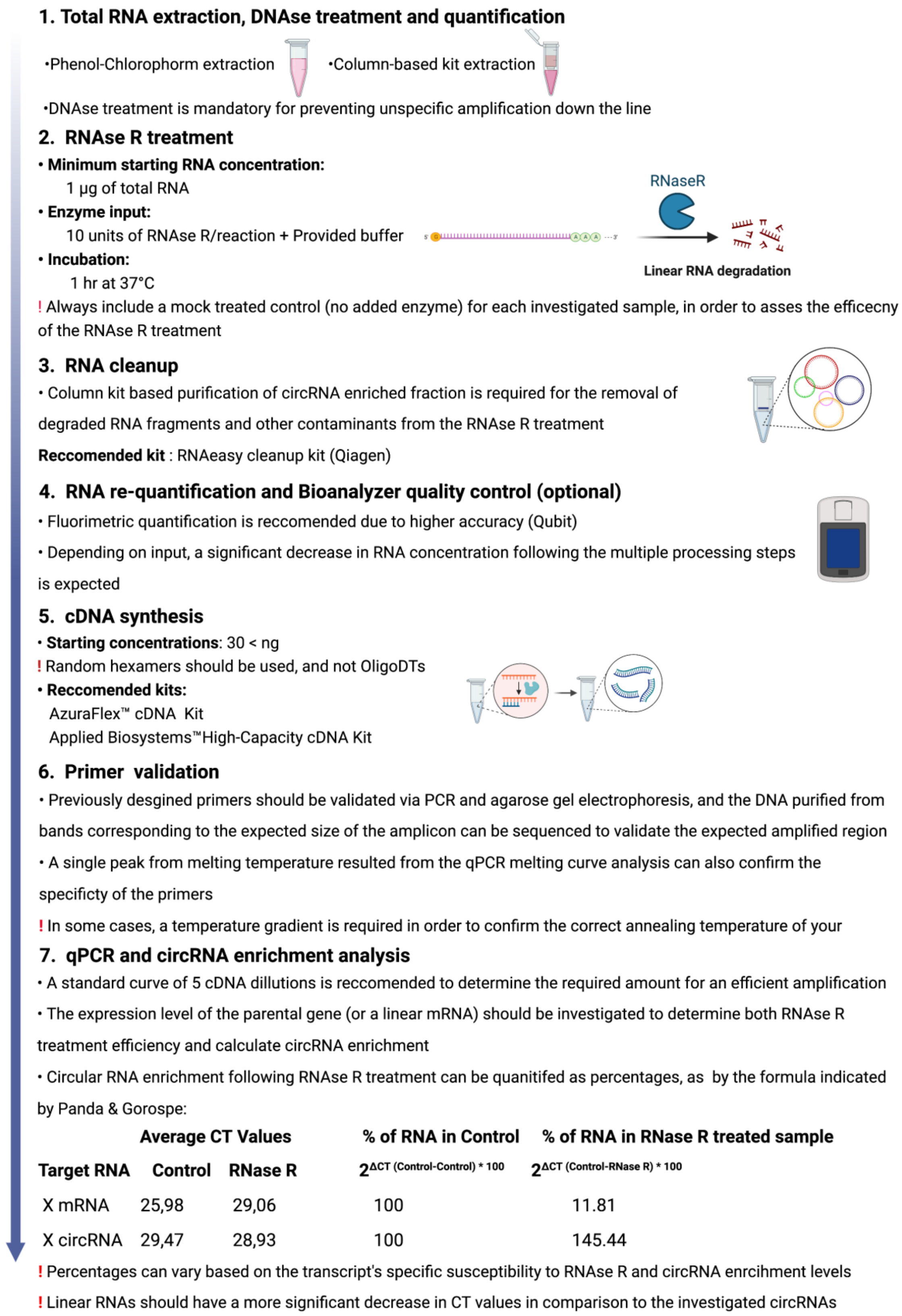

Supplemental Figure 1. Detailed step-by-step protocol for the techniques and steps required for the analysis of circRNA using qPCR

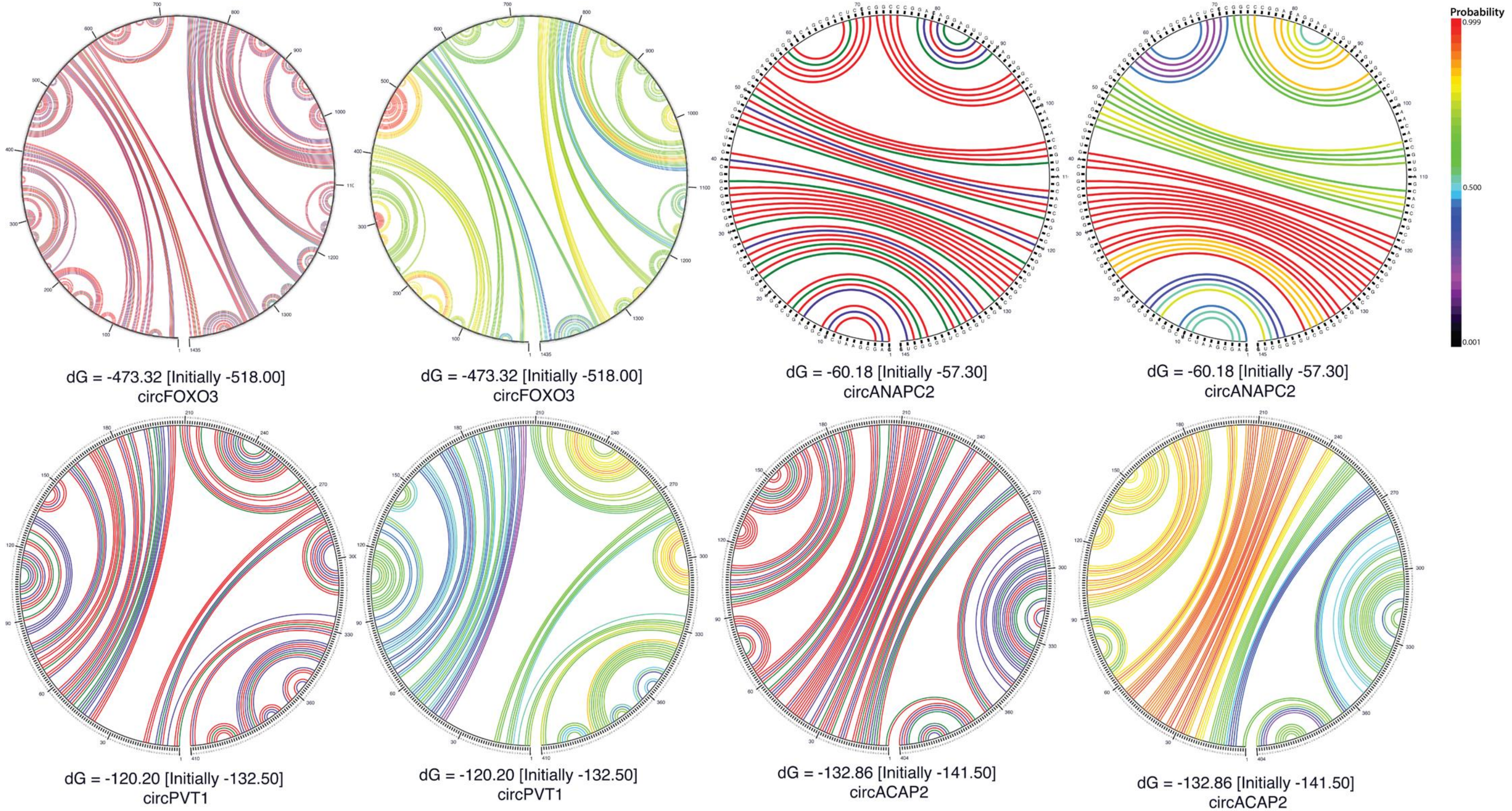

Supplemental Figure 2. Secondary structure formation prediction based on nucleotide complementarity (left, for each circRNA), the thermodynamic probability of their interaction (right, legend for interaction probability on far right) and calculated standard free energies (dG)

Supplemental Table 1. Nucleotide and GC% composition of the circRNAs.

Transcript	Total Bases	A	C	G	U	GC%
**circFOXO3**	1435	333	441	369	292	56,4
**circPVT1**	410	90	92	116	112	50,7
**circANAPC2**	145	20	37	65	23	70,3
**circACAP2**	410	82	104	125	93	56,7

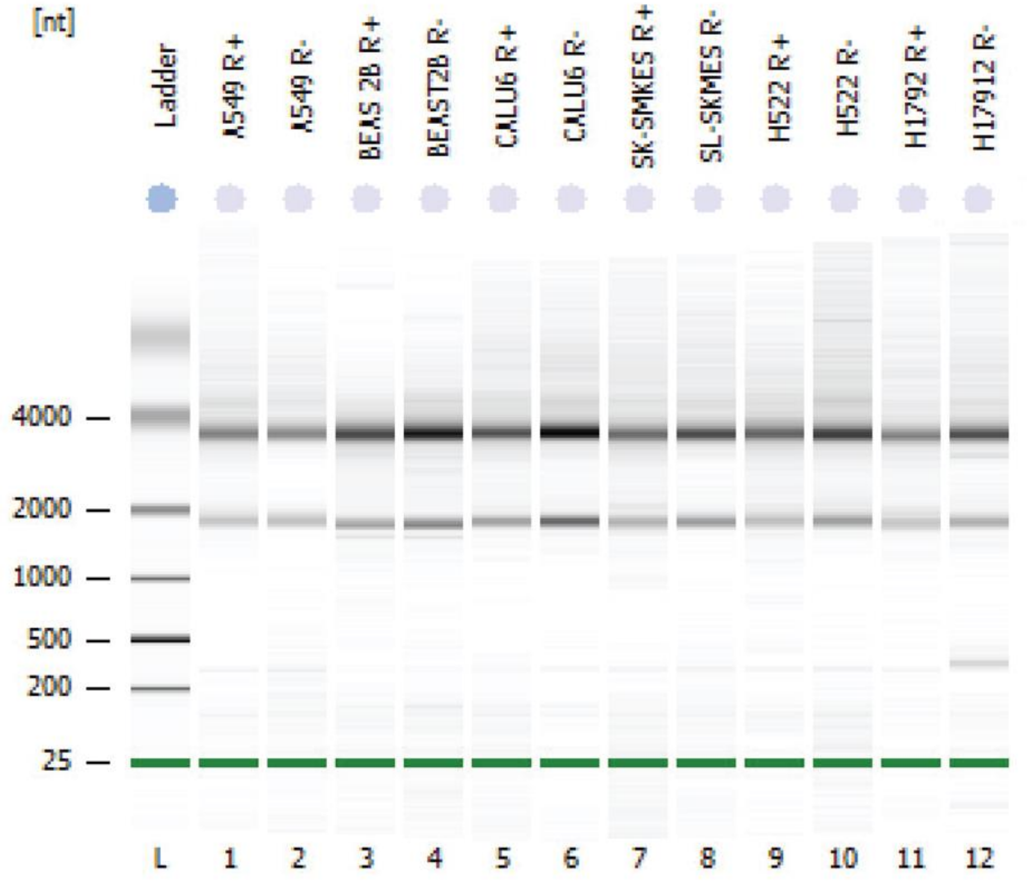

Supplemental Figure 3. Bioanalyzer electrophoresis gel summary of RNAse R treated and untreated samples after cleanup

Supplemental Table 2. Primers utilized in the study

Circular RNA	Accession	Amplicon (bp)	Regions	Forward	Reverse
circFOXO3	NM_001455	183	Exon 2	GTGGGGAACTTCACTGGTGCTAAG	GGGTTGATGATCCACCAAGAGCTCTT
circPVT1	NR_003367	118	Exon 2	CTCTTCCTGGTGAAGCATCTG	CTGACAGGCACAGCCATCT
circANAPC2	NM_013366	162	Exon 3-1	AGAGGATGGAGGACCGTTG	TCCAGGCCACTAACAACTCC
circACAP2	NM_012287	229	Exon 3-1	TAATGATGCTGTCGTTGAGC	CCTTCAGACACTCCTCGAAA
FOXO3	NM_001455			GCAAGAGCTCTTGGTGGA	TGGGGCTGCCAGGCCACTTGGAGAG
PVT1	NR_003367			TGGAATGTAAGACCCCGACTCT	GATGGCTGTATGTGCCAAGGT
ANAPC2	NM_013366			TATGTTGCGCGGAGTCTTGT	CCAGCACTGTTGCTTGTCAC
ACAP2	NM_012287			TCCAGGAGCCGCACTTCTA	CTCCGGGATGTGGATCTTCA

GAPDH	NM_002046	GTCTCCTCTGACTTCAACAGCG	ACCACCCTGTTGCTGTAGCCAA

Supplemental Table 3. Sample RNA concentrations following processing steps

Sample Characteristics			Concentration after RNAse Treatment and Cleanup				
Nr.	Name	Starting Concentration (ng/uL)	RNAse R	Nanodrop (ng/uL)	Qbit (ng/uL)	RIN	cDNA Input
1	A549	332	+	30.2	9,2	9.70	100
2	A549		-	10,2	3,4	9.4	
3	BEAS2B	220	+	30,4	11,26	9.2	
4	BEAS2B		-	13.2	4,4	9.9	
5	CALU6	566	+	30.6	3,76	9.9	
6	CALU6		-	10.3	2,94	10	
7	SK-MES1	272	+	29.1	3,4	9.80	
8	SK-MES1		-	9,6	4,94	10	
9	H522	174	+	27	7,96	8.8	
10	H522		-	11,2	2,64	10	
11	H1792	240	+	33.3	10,2	9.40	
12	H1792		-	10,3	1,4	8.40	

Supplemental Table 4. Exemplified method of calculating post-RNAse R enrichment of circRNAs and linear transcripts based on averaged CT values derived from the qPCR analysis of A549 RNAse R treated and untreated control RNA samples

		Average CT values		% of RNA in Control	% of RNA in RNase R
**Sample**	**Target RNA**	**Control (RNAse R-)**	**RNase R+**	**2^ΔCT (Control-Control) * 100**	**2^ΔCT (Control-RNase R) * 100**
A549	FOXO3	28,367	33,186	100%	3,54%
	circFOXO3	29,133	29,050		105,88%
	PVT1	26,984	33,125		1,42%
	circPVT1	33,033	31,268		340,06%
	ANAPC2	26,562	31,968		2,36%
	circANAPC2	28,735	28,490		118,53%
	ACAP2	29,995	30,820		56,42%
	circACAP2	31,562	31,831		83,00%

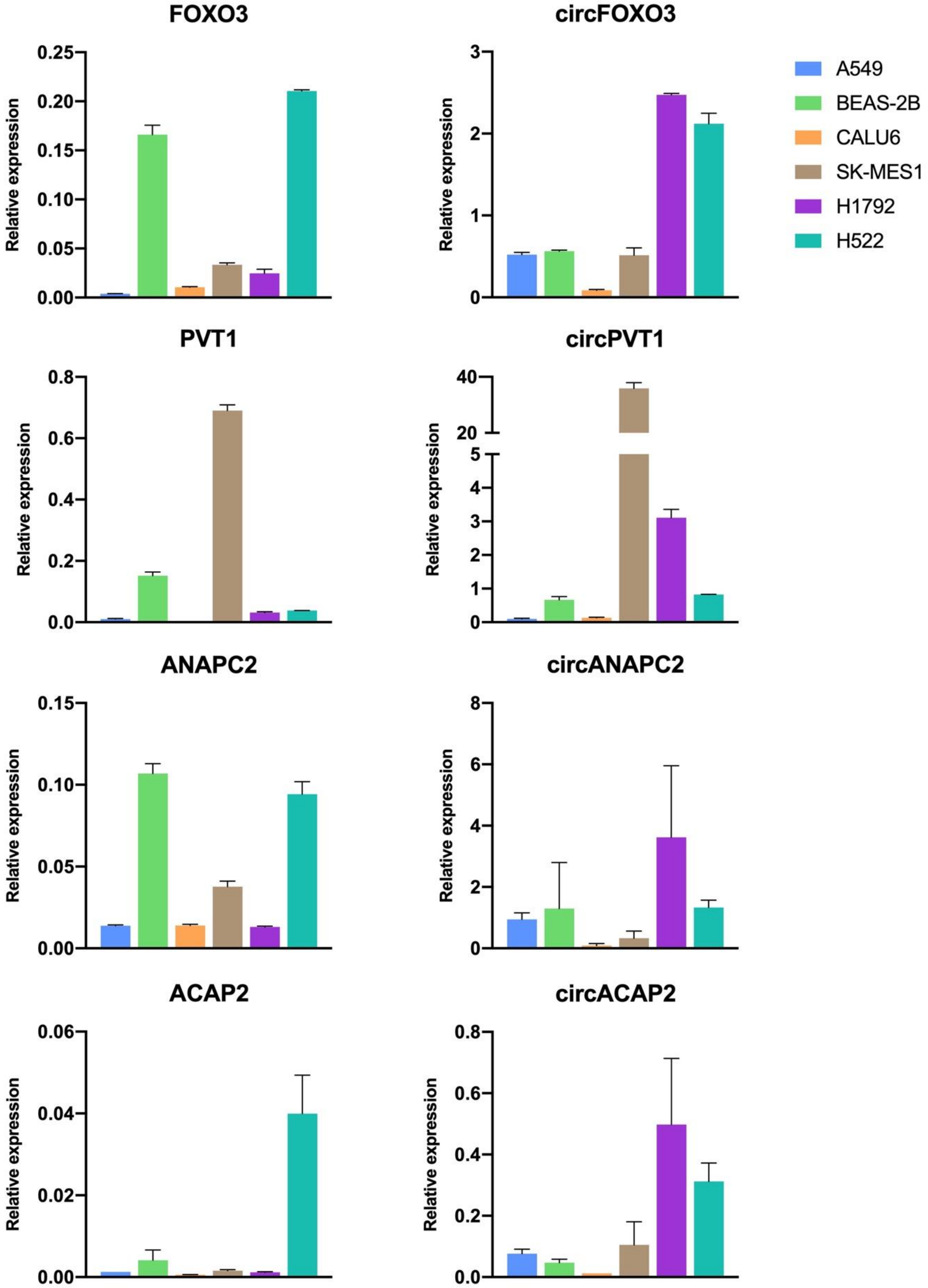

Supplemental Figure 4. Comparative relative expression levels of parental genes (left) and circRNAs (right) in the investigated cell lines.

Supplemental Table 5 Name and characteristics of the utilized circRNA databases utilized in the study

	Name	circRNA Annotation	Primer Design Tool	miRNA Binding	RBP Binding	ORF Information	Reference	Comments
1	circBank	×	-	×	-	×	[33]	Offers information about circRNA conservation and m6a modifications
2	circBase	×	-	-	-	-	[32]	Extensive; Includes main curated circRNA datasets available; Provides scripts to identify circRNA in sequencing data
3	circInteractome	circBase annotation	×	×	×	-	[44]	Extensive tool-wise; Includes both divergent primer and siRNA design tools
4	EncoRI (starbase)	circBase annotations	-	×	×	-	[41]	Database focused on miRNA and lncRNA interactions; circRNA-miRNA interactions based on AGO CLIP-seq Data
5	Circ2GO	Genomic coordinates	-	×	-	-	[43]	Contains RNAseq data from 60 human lung cell lines and 50 lung adenocarcinoma (LUAD) cell lines. ; Transcript map and expression level heatmaps generating tools
6	CSCD	Genomic coordinates	-	×	×	×	[42]	Detailed information about circRNA structure, cellular localization, and alternative splicing influence on circRNA composition.

"×" - yes; "-"- no;
